# Can therapeutic potency of a cancer nanomedicine be predicted by pain-related behavioral test in subcutaneous tumor model?

**DOI:** 10.1016/j.ijpx.2025.100450

**Published:** 2025-11-17

**Authors:** Ye Tao, Xiaohui Cai, Zhongping Chen

**Affiliations:** a Medical School, Nantong University, Nantong, PR China; b Institute of Special Environmental Medicine, Nantong University, Nantong, PR China; c Department of Hematology, The Third Affiliated Hospital of Nanjing Medical University, Changzhou, PR China

**Keywords:** Lipid nanoparticles, Nanomedicine, Subcutaneous tumor, Cancer pain, Behavioral test, Therapeutic evaluation

## Abstract

Cancer nanomedicines have shown great potential in fighting against cancer. While the development of cancer nanomedicines is advancing rapidly, preclinical assessment approaches for their therapeutic potency have stagnated. In view of high prevalence of cancer pain in cancer patients, we aim to determine whether therapeutic potency of a cancer nanomedicine can be predicted by pain-related behavioral test in subcutaneous tumor model, the simplest and most widely used tumor model in oncology. Behavioral profiles reveal that subcutaneous tumor, probably irrespective of tumor type, presents with spontaneous pain (open field test) and evoked pain (von Frey test for mechanical allodynia; Hargreaves test, hot plate test, and tail flick test for thermal hyperalgesia; cold plate test and acetone drop test for thermal allodynia). Using doxorubicin (DOX)-loaded lipid nanoparticles (LNPs) (LNPs/DOX) as a representative cancer nanomedicine and ropivacaine (ROP)-loaded LNPs (LNPs/ROP) as a pain nanomedicine, it is validated that inhibiting subcutaneous tumor growth can relieve cancer pain, while delaying the growth cannot, despite a significant difference found compared with non-treatment group. Moreover, behavioral results in all the tests are consistent and von Frey test is suggested the most sensitive among them. It is strongly suggested that pain-related behavioral test can serve as a powerful tool to predict therapeutic potency of a cancer nanomedicine *in vivo* in treating subcutaneous tumor.

## Graphical abstract

**Figure:**
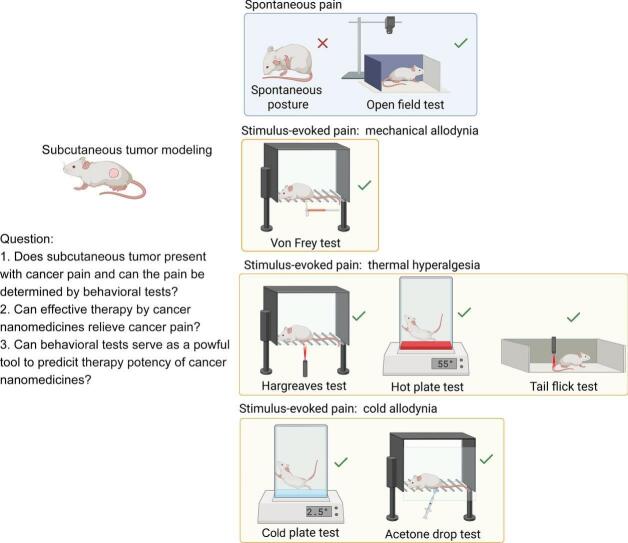

## 1 Introduction

Globally, more than 20 million new cases of cancer were diagnosed in 2022 and the incidence is continuously rising with new cases estimated to reach over 35 million by 2050 (Bray et al., 2024). Cancer continues to pose a formidable challenge to global health, with its complex nature making it difficult to treat. Over recent decades, great progress has been made in the fight against cancer, driven by the convergence of nanotechnology with fields, such as biology, material, chemistry, biomedical engineering, and pharmacology. These interdisciplinary efforts have led to the development of nanoparticle-based cancer nanomedicines to treat various types of cancer. Extensive preclinical and clinical data have demonstrated that cancer nanomedicines offer several key benefits:1) improved pharmacokinetic profiles of bioactive compounds by enhancing their solubility, tailoring their release dynamic, and shielding them from degradation when circulating in the blood; 2) increased concentration of therapeutic agents in tumor tissue through passive or active targeting mechanism; 3) improved cell uptake *via* nanoparticle-mediated endocytosis; 4) mitigated adverse effects associated with free chemotherapeutic drugs (Henn et al., 2024; Ullah et al., 2025). By utilizing these benefits, cancer nanomedicines demonstrate therapeutic superiority over traditional chemotherapy. Recent innovations in nanotechnology have further expanded their potential by endowing them with some novel features:1) stimuli-responsive or transformable properties and biomimetic designs; 2) combination strategy with other treatments, including multidrug chemotherapy, radiotherapy, photodynamic therapy, photothermal therapy, and immunotherapy (Kanungo et al., 2025). On the other hand, although the development of cancer nanomedicines is advancing rapidly, assessment approaches for their therapeutic potency have stagnated, which are limited to monitoring tumor growth and survival of model animals *in vivo* as well as assessing pathological and immunological changes of tumor tissues *ex vivo*.

Pain is one of the most common symptoms reported by cancer patients, which exerts a severe impact on quality of life and is associated with numerous psychosocial responses. Cancer pain encompasses complicated and poorly understood mechanisms, resulting from successive changes in cellular, tissue, and systemic levels. Cancer itself can directly cause pain through mechanical compression, release of algogenic substances, and nerve invasion. Meanwhile, cancer treatments, such as chemotherapy, radiotherapy, immunotherapy, and surgery, can also give rise to iatrogenic pain. As a result, cancer pain may be nociceptive and neuropathic. Diverse causes and mechanisms intertwine with each other, making cancer pain prevalent and persistent (Bohren et al., 2025; Mulvey et al., 2024). A recent meta-analysis collecting the literature published from January 2014 to December 2021 revealed that the overall prevalence of pain in cancer patients was 44 % and pain prevalence in advanced cancer was 55 % (Snijders et al., 2023). In view of the high prevalence of cancer pain in patients, several cancer pain models are established in animals. The most successfully model is bone cancer pain. Studies from others and ours verified that treating bone cancer by cancer nanomedicines could relieve pain (Gu et al., 2024; Long et al., 2021). Some non-bone cancer pain models, such as pancreatic cancer, squamous cell carcinoma, and neuroma, are also reported. However, they are confined to mechanistic study, which aims at studying pain rather than treating cancer (Haroun et al., 2023).

Among a broad range of tumor models, subcutaneous model is the simplest, involving cancer cells or tissues from mouse or human growing beneath the skin of a mouse. Despite the recognition that it lacks complex microenvironment and interaction found in original tissue, it by far remains the most widely used in basic and translational oncology, due to its low cost, high availability, easy establishment, and short time of tumor development (Stribbling and Ryan, 2022). As various cancer nanomedicines have demonstrated success, it is an interesting topic to discuss whether pain-related behavioral test can serve as a universal tool for assessing therapeutic potency of cancer nanomedicines in subcutaneous tumor model-involved preclinical studies, which might ultimately benefit their translation from bench to bedside. We thus prepared two kinds of nanomedicines, a cancer nanomedicine of lipid nanoparticles (LNPs) with a conventional chemotherapeutic agent (doxorubicin, DOX) as the payload and a pain nanomedicine of LNPs with a conventional analgesic (ropivacaine, ROP) as the payload, to address the following concerns: 1) Does subcutaneous tumor present with pain? 2) As there is no well-established behavioral methodology to determine non-bone cancer pain, can the pain be determined by behavioral tests available for bone cancer pain as well as other pain conditions? 3) Among various tests, which is more suitable to reflect the pain? 4) Can effective treatment of subcutaneous tumor relieve the pain?

## 2 Materials and methods

### 2.1 Materials

1,2-Dioleoyl-*sn*-glycero-3-phosphoethanolamine (DOPE), 1,2-distearoyl-*sn*-glycero-3-phosphoethanolamine-polyethylene glycol-2000 (DSPE-PEG), and cholesterol were purchased from AVT (Shanghai) Pharmaceutical Tech Co. Ltd. DOX and ROP were obtained from Aladdin Scientific Corp. (Shanghai, China). All other chemicals were from Sinopharm Chemical Reagent Co. Ltd. (Shanghai, China) and used as received.

### 2.2 LNPs synthesis and characterization

LNPs were synthesized based on our previous work (Huang et al., 2024). In a typical experiment, DOPE, cholesterol, and DSPE-PEG (7:2:1, molar ratio) were dissolved in anhydrous ethanol and then mixed with water (ethanol: water = 1/3, *v*/v). The mixture was dialyzed against water (MWCO = 14,000) for 3 h under gentle magnetic stirring to generate LNPs. To load DOX or ROP into LNPs, DOX or ROP was mixed with all lipids (1/4, *w*/w), which was then subjected to dialysis, according the above-described protocol, to produce LNPs/DOX or LNPs/ROP. LNPs/DOX and LNPs/ROP were lyophilized prior to intravenous use.

Transmission electron microscopy (TEM, JEM-1230, Japan) was used to observe the size and morphology of LNPs. Dynamic light scattering (DLS, Zetasizer ZS90, Malvern) was utilized to determine the hydrodynamic size, polydispersity index (PDI), and zeta potential of LNPs.

### 2.3 Drug quantitation

High performance liquid chromatography (HPLC, Waters) was used to quantify DOX or ROP. The mobile phase for DOX was methanol/acetonitrile/0.02 M NH_4_H_2_PO_4_/glacial acetic acid (52:5:43:6, *v*/v) (Weng et al., 2025), while it was 0.02 M KH_2_PO_4_/acetonitrile (65:35, *v*/v) for ROP. Detection wavelengths were and 488 and 210 nm for DOX and ROP, respectively. The other HPLC parameters for DOX and ROP were the same: chromatographic column, ultimate®AQ-C_18_; flow rate, 1 mL/min; column temperature, 30 °C; injection volume, 20 μL.

To determine DOX or ROP content in LNPs for drug loading efficiency and drug loading capacity assessment, LNPs/DOX or LNPs/ROP suspension was treated with methanol (1/20, v/v), followed by sonication to crumble LNPs. After centrifugation at 8000 rpm for 10 min, the supernatant was collected to filter with 0.45 μm organic membrane. Loaded drug in filtrate was determined by HPLC. Drug loading efficiency was calculated by normalizing loaded drug relative to initially fed drug (*w*/w); drug loading capacity was calculated by normalizing loaded drug relative to loaded drug plus total lipids (w/w).

In another experiment to assess *in vitro* drug release from LNPs, 1 mL of LNPs/DOX or LNP/Rop was dispersed in 10 mL of PBS (pH = 7.4) with gentle shaking. At the predetermined timepoints (0, 0.05, 0.25, 0.5, 1, 2, 4, 6, 12, and 24 h), 100 μL of solution was withdrawn and treated with 1.9 mL of methanol for HPLC analysis.

### 2.4 Subcutaneous tumor modeling and postoperative subcutaneous tumor modeling

Mouse breast cancer cell (4 T-1) line (Institute of Biochemistry and Cell Biology, Shanghai, China) was cultured in RPMI-1640 with supplemented 10 % fetal bovine serum (FBS) and 1 % antibiotics in a humidified CO_2_ (5 %) atmosphere. Cells were collected and the suspension was placed on ice prior to tumor modeling.

BALB/c mice (6–8 weeks old, female) were supplied by Animal Center of Nantong University. All animal experiments were approved by the Animal Care and Use Committee of Nantong University (Approval ID: S20241202–003) and the Jiangsu Province Animal Care Ethics Committee (Approval ID: SYXK[SU] 2007–0021). BALB/c mice were subcutaneously injected with 100 μL of 4 T-1 cells (5 × 10^6^) in the left back with the left hind leg not being affected. To establish postoperative subcutaneous tumor modeling, around 80 % of tumor was removed by surgery. Tumor volume was recorded and calculated as tumor volume = (length × width^2^)/2. Mice were placed in an animal room and given free access to food and water throughout the experiment. The animal study was carried out in accordance with the ARRIVE Essential 10 checklist.

### 2.5 Grouping and drug treatment

There were 6 groups (*n* = 8): 1) a group bearing subcutaneous tumor, receiving PBS treatment; 2) a group bearing subcutaneous tumor, receiving LNPs/DOX treatment; 3) a group bearing subcutaneous tumor, receiving LNPs/ROP treatment; 4) one group bearing postoperative subcutaneous tumor, receiving PBS treatment; 5) one group bearing postoperative subcutaneous tumor, receiving LNPs/DOX treatments; 6) particularly, a healthy group set to get baseline (BL) data and then receiving LNPs/DOX treatment to determine whether LNPs/DOX treatment will result in chemotherapy-induced neuropathic pain (CINP). Mice receiving intravenous treatment of LNPs/DOX and LNPs/ROP at 5 mg DOX and 3 mg ROP/kg body weight. For LNPs/ROP, behavioral test was performed at 24 h post treatment, suggested by its duration of action. Following the last behavioral test, all mice were euthanized using CO_2_. Detailed protocols were illustrated in Results and discussion section.

### 2.6 Spontaneous pain

#### 2.6.1 Spontaneous posture

Spontaneous posture, such as paw lifting and flinching, was tested based on our previous work (Long et al., 2021). Tumor-bearing mice were placed in a plastic box where their movement was unrestricted. The number of flinches and the cumulative duration of lifting of the hind paw ipsilateral to tumor modeling site was recorded over a period of 4 min. Increased number of flinches and duration of lifting indicate spontaneous pain.

#### 2.6.2 Open field test

Tumor-bearing mice were placed in the center of a circular arena surrounded by fences, and then allowed for free movement. The activities of mice, including the mean speed, total distance traveled, time spent in the central area, and distance in the central area, were recorded using automated tracking software. Reduced distance, speed, and time reflect spontaneous pain.

### 2.7 Stimulus-evoked pain

#### 2.7.1 Von Frey test for mechanical allodynia

Von Frey test was performed with the protocol detailed in our previous work (Huang et al., 2024), which was developed by Dixon (Dixon, 1980). Briefly, a series of calibrated von Frey filaments with varying bending forces were used to stimulate the plantar surface of the hind paw ipsilateral to tumor modeling. The minimal force required to elicit a withdrawal response (*e.g.*, paw fluttering, licking, and lifting) was recorded as paw withdrawal threshold (PWT). Reduced threshold indicates mechanical allodynia.

#### 2.7.2 Hargreaves test, hot plate test, and tail flick test for thermal hyperalgesia

With the protocol pioneered by Hargreaves (Hargreaves et al., 1988), the plantar surface of the paw of mice was exposed to a focused heat beam. The time taken to withdraw the paw was recorded as paw withdrawal latency (PWL). For hot plate test, mice were placed on a heated metal plate (55 °C) and the latency to display withdrawal response (*e.g.*, paw jumping and shaking) was recorded as PWL. For tail flick test, mice were restricted in a holder and a focused beam was applied to heat the tail. The time taken for tail withdrawal was recorded as tail withdrawal latency (TWL). In these three tests, a cutoff time (15–30 s) needs setting to prevent tissue damage and reduced latency reflects thermal hyperalgesia.

#### 2.7.3 Cold plate test and acetone drop test for cold allodynia

The protocol for cold plate test is similar to that for hot plate test. Mice were placed on a cold metal plate (2.5 °C) and the latency to display withdrawal response was recorded as PWL. A cutoff time of 60 s was set. Reduced latency reflects cold allodynia.

For acetone drop test, a drop of acetone (100 μL) was applied to the plantar surface of the hind paw using a pipette. The response was recorded and graded to the following 4-point scale: 0, no response; 1, rapid withdrawal, flicking, or stamping of the paw; 2, prolonged withdrawal or repeated flicking of the paw; 3, repeated flicking of the paw with licking (Kukkar et al., 2014). Acetone was applied three times with an interval of 5 min to get an average score. Elevated score indicates cold allodynia.

### 2.8 Statistical analysis

Data were statistically analyzed using unpaired student's *t*-test for two-group comparison and one-way analysis of variance (ANOVA) test for multiple-group comparison, respectively, in GraphPad Prism 8.0. A *p*-value less than 0.05 (*p <* 0.05) was considered significantly different. Data were expressed as mean ± standard deviation (SD) for *in vitro* results or mean ± standard error of the mean (SEM) for *in vivo* results.

## 3 Results and discussion

### 3.1 Synthesis and characterization

Currently, there are diverse nanoparticles, such as liposomes, polymeric micelles, and a wide spectrum of inorganic nanoparticles, serving as the carrier of nanomedicines to manage diseases. Among them, liposomes assembled from lipids are the most successful carrier, due to their advantages in good biocompatibility and well-established profiles in synthesis and drug loading, leading to the fact that most of approved cancer nanomedicines are liposomes (Chen et al., 2024; Gatto et al., 2024). Meanwhile, liposomes also play a great role in pain nanomedicines (Xu et al., 2023). Liposomal bupivacaine (Exparel) and liposomal morphine (DepoDur), which account for 40 % of approved pain nanomedicines (2/5), have been widely used for pain management (Torpey et al., 2024). LNPs, compositionally similar to but structurally different from liposomes, have been attracting increasing interest, inspired by their great success in battling COVID-19 pandemic. Particularly, our recent findings demonstrated the therapeutic superiority of LNPs over liposomes in treating cancer, realized by their relatively high structural stability and rigidity for improved pharmacokinetics and cell uptake (Huang et al., 2024). LNPs are hence selected as the carrier of DOX and ROP.

**Fig. 1:**
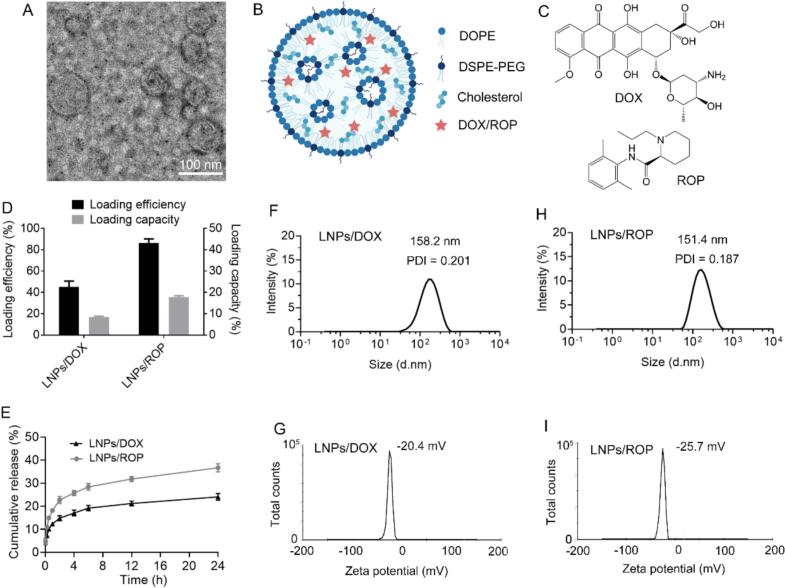
Synthesis and characterization of LNPs. (A) Representative TEM image of blank LNPs. (B) Schematic illustration for the structure of LNPs. (C) Molecular structure of DOX and ROP; (D) Drug loading efficiency and capacity of LNPs/DOX and LNPs/ROP. (E) *In vitro* release curves of LNPs/DOX and LNPs/ROP within 24 h. (F—I) Hydrodynamic size and zeta potential of LNPs/DOX and LNPs/ROP. Data are presented as mean ± SD (*n* = 3).

LNPs are formulated, using DOPE as main lipid, cholesterol to stabilize the structure of LNPs, and DSPE-PEG as helper lipid to extend blood circulation time, through ethanol dilution method. Fig. 1A exhibits representative TEM image of blank LNPs. It is observed that LNPs are spherical with the size less than 80 nm in dried state. It is noted that the exact structure of LNPs still remains unclear, which might vary with the kind of lipids, lipid combination, and payload. Generally, they are suggested to have a core composed of multiple inverted micelle-like lipid compartments, which is surrounded by a lipid monolayer (Szebeni et al., 2023; Wang et al., 2025). Hydrophobic DOX and ROP are encapsulated into the continuous lipid phase inside LNPs. Suggested structure of LNPs is schematically illustrated in Fig. 1B and C. Drug loading efficiency and capacity of LNPs are also investigated. As shown in Fig. 1D, DOX loading in LNPs is unsatisfactory with the drug loading efficiency less than 50 % and corresponding drug loading capacity around 8.4 %. Moreover, DOX cannot be efficiently loaded into DOPE-based liposomes, irrespective of whether it is hydrophobic or hydrophilic (data not shown). As our previous work reported that DOX could be successfully loaded into 1,2-dipalmitoyl-sn-glycero-3-phosphorylcholine (DPPC), 1,2-dimyristoyl-sn-glycero-3-phosphocholine (DMPC), and 1-palmitoyl-2-stearoyl-sn-glycero-3-phosphocholine (HSPC)-based liposomes (Gu et al., 2024; Li et al., 2019; Long et al., 2020), the unsatisfactory DOX loading herein should not result from the structure of LNPs. There is a repulsive force probably existing between DOX and DOPE, contributing to such a phenomenon. The reason deserves further investigation, which is however not the concern of this work. Of course, other hydrophobic chemotherapeutic agents, for example, taxanes (paclitaxel and its derivative, docetaxel), may be alternatives (Zhou et al., 2021). However, they themselves can definitely induce CINP (Lee et al., 2024) and are thus not selected.

Comparatively, the loading of ROP into LNPs is successful, showing a loading efficiency of 86.3 % and a loading capacity of 17.8 %. *In vitro* release profiles display that as expected, LNPs can retain their payloads well with less than 40 % of the payloads released within 24 h and particularly, LNPs retain DOX better than ROP, because of the relatively big molecular weight of DOX (Fig. 1E). Both LNPs/DOX and LNPs/ROP belong to sustained-release formulations. DLS results show that LNPs/DOX and LNPs/ROP have a similar hydrodynamic size (158.2 nm for LNPs/DOX and 151.4 nm for LNPs/ROP); the zeta potential of LNPs/DOX is slightly higher than that of LNPs/ROP, due to the fact that DOX is a cationic drug (Fig. 1F-I).

### 3.2 Subcutaneous tumor presents with pain

**Fig. 2:**
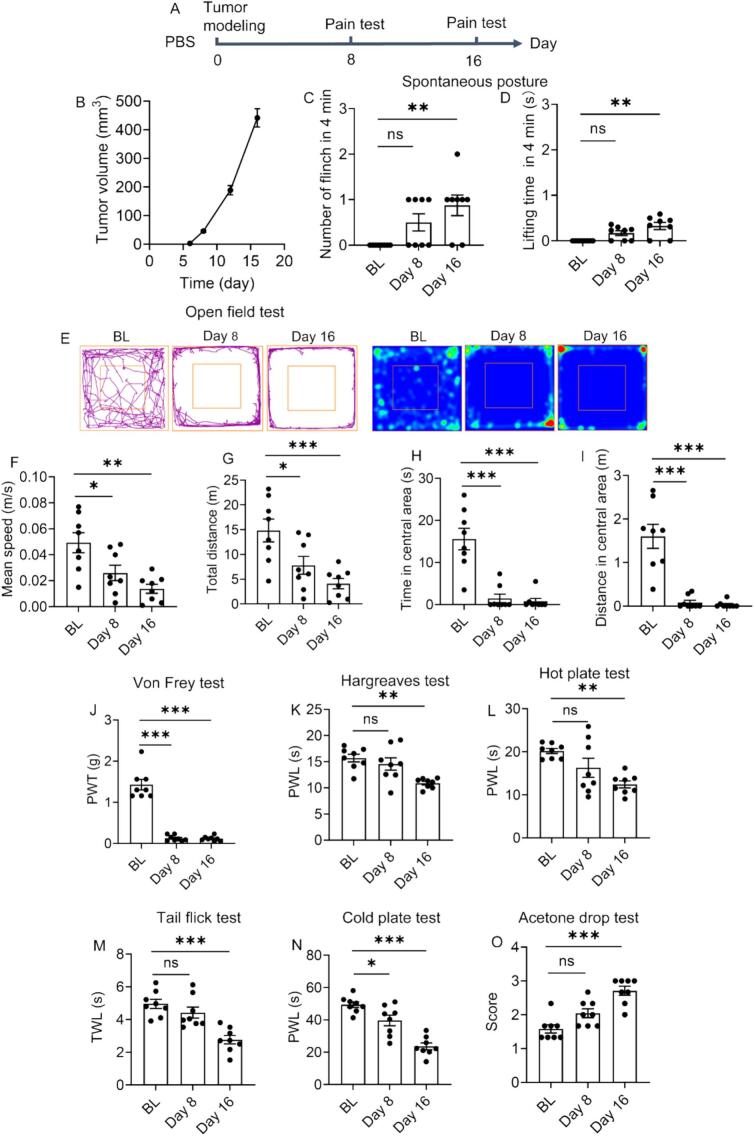
Behavioral test of tumor-bearing mice receiving PBS treatment. (A) Timepoint for behavioral test. (B) Tumor growth monitoring. Spontaneous posture test by (C) number of flinch and (D) lifting time of the hind paw ipsilateral to tumor modeling site in 4 min. (E) Movement trajectory in open field test and corresponding pseudo-color image. (F) Mean speed, (G) total distance, (H) time spent in central area, and (I) distance in central area analyses in open field test. Stimulus-evoked pain evaluation by (J) von Frey test, (K) Hargreaves test, (L) hot plate test, (M) tail flick test, (N) cold plate test, and (O) acetone drop test. Data are presented as mean ± SEM (*n* = 8). BL data come from healthy mice. *p < 0.05, **p < 0.01 and ***p < 0.001; ns = not significant.

Cancer pain patients suffer nociceptive pain (60 %), neuropathic pain (20 %), or a combination of them (20 %), presenting with spontaneous and stimulus-evoked pain (Money and Garber, 2018). Compared with evoked pain, spontaneous pain is more realistic, as it is a daily experience of patients. Spontaneous pain is often discussed in the context of inflammatory and neuropathic pain conditions. Despite its distinction from inflammatory and neuropathic pain, compelling evidence validate the signs of spontaneous pain in rodent cancer pain models (Mravec, 2022). The measurement of spontaneous pain in rodents is challenging, which is a multiplex issue, including spontaneous posture, ultrasound vocalization, burrowing behavior, rat grimace scale, gait analysis, and conditioned preference test (Ma et al., 2022). Encouraged by the success in determining bone cancer pain, spontaneous posture was first utilized to determine spontaneous pain of subcutaneous tumor. With the protocol illustrated in Fig. 2A, subcutaneous tumor model is successfully established and the number of flinch and the duration of lifting of the hind paw ipsilateral to tumor modeling are found to slightly increase (Fig. 2B-D). Although a statistical difference is observed along with tumor growth, the change is minor (<1 in the mean number of flinch and < 0.35 s in the mean lifting time. As bone cancer could result in a huge change in spontaneous posture (>10 in mean number of flinch and > 2 s in mean lifting time) indicated in our previous work (Long et al., 2021), this negligible change herein is believed to result from the hindrance of tumor growth to the use of the paw rather than pain. Thus, spontaneous posture is considered unsuitable for assessing spontaneous pain caused by subcutaneous tumor and is not adopted in the later experiments. In this context, gait analysis recording weight bearing of paw is also not suitable. Ultrasound vocalization, burrowing behavior, rat grimace scale, and conditioned preference test might be promising approaches, which are however not carried out, due to the lack of apparatus. Open field test is commonly used to evaluate pain-induced anxiety. On the other hand, as open field test also reports exploratory behavior and locomotion activity, it can serve as the signs of spontaneous pain (Huerta et al., 2024). As shown in Fig. 2E-I, the mean speed, total distance traveled, time spent in the central area, and distance in the central area in open field significantly decrease with a tendency to deteriorate over time, indicative of persistent spontaneous pain.

Apart from ongoing spontaneous pain, a minor noxious or non-noxious stimulus (mechanical, thermal, and cold stimuli) can evoke cancer pain. Stimulus-evoked pain can present as allodynia, a pain elicited by a stimulus that does not cause pain, and hyperalgesia, an aggravated pain evoked by a stimulus usually causing pain, which are commonly used in preclinical research to assess pain behaviors in nociceptive and neuropathic pain (Modi et al., 2023). In this work, mechanical allodynia (von Frey test), thermal hyperalgesia (Hargreaves test, hot plate test, and tail flick test), and cold allodynia (cold plate test and acetone drop test) were utilized. The results reveal that subcutaneous tumor significantly reduces pain threshold and latency as well as elevating pain score (Fig. 2J-O), indicating that subcutaneous tumor can induce evoked pain and the pain can be assessed by mechanical, thermal, and cold allodynia/hyperalgesia. Moreover, it is found that von Frey test might be the most sensitive among all tests. According to the tumor growth profile, the tumor size on the 8th day is only 45.6 mm^3^ (Fig. 2B) and however, it can cause severe mechanical allodynia, with the pain threshold sharply decreasing from 1.3 g of BL to 0.12 g (*p <* 0.001). This result also strongly suggests that the generation of cancer pain of subcutaneous tumor does not merely rely on tumor size. Comparatively, thermal and cold allodynia/hyperalgesia are not evident on the 8th day, although a tendency is found, and a significant difference occurs on the 16th day, where there is a considerable tumor growth (441.5 mm^3^). It is suggested by others that von Frey test can be used for any type of rodent pain model; only von Frey test as well as thermal preference tests can measure cancer pain, while thermal and cold allodynia/hyperalgesia-involved tests, are best suited for inflammatory, neuropathic, arthritic, and muscle pain models (Modi et al., 2023). While corroborating the effectiveness and sensitivity of von Frey test, our findings demonstrate that thermal and cold allodynia/hyperalgesia-involved tests can be also suitable for cancer pain measurement, despite a relatively low sensitivity. The behavioral profiles collectively demonstrate that: 1) subcutaneous tumor presents with both spontaneous and evoked pain; 2) the pain can be assessed by well-established approaches, except the tests recording spontaneous behavior of paw; 3) von Frey test shows particular advantage in sensitivity.

### 3.3 Delaying subcutaneous tumor growth cannot relieve cancer pain

**Fig. 3:**
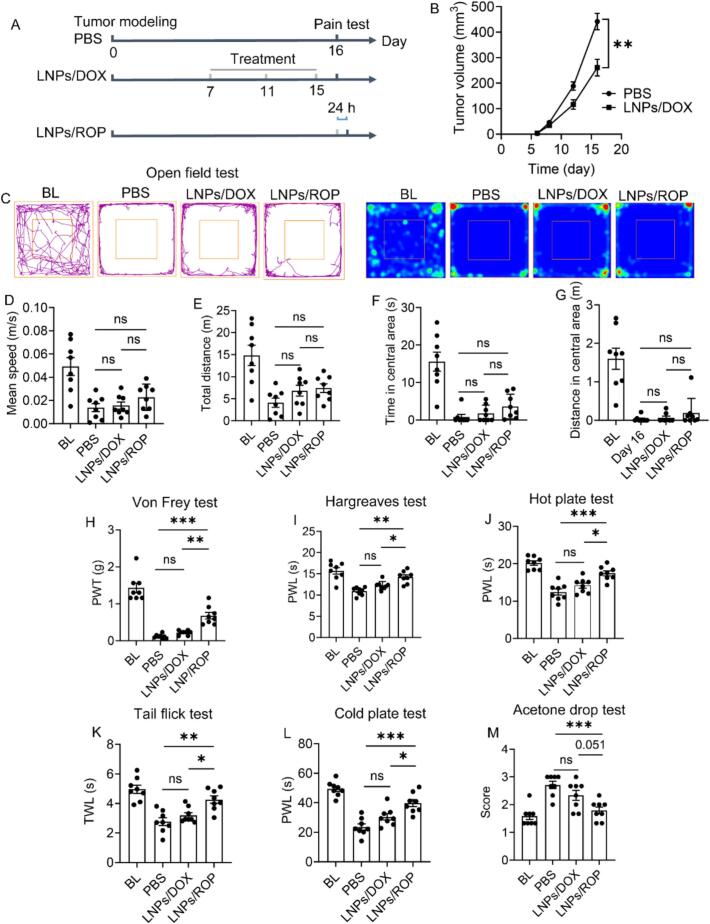
Behavioral test of tumor-bearing mice receiving PBS, LNPs/DOX, and LNPs/ROP treatments. (A) Timepoint for drug treatment and behavioral test. (B) Tumor growth monitoring. (C) Movement trajectory in open field test and corresponding pseudo-color image. (D) Mean speed, (E) total distance, (F) time spent in central area, and (G) distance in central area analyses in open field test. Stimulus-evoked pain evaluation by (H) von Frey test, (I) Hargreaves test, (J) hot plate test, (K) tail flick test, (L) cold plate test, and (M) acetone drop test. Data are presented as mean ± SEM (*n* = 8). BL data come from healthy mice. *p < 0.05, **p < 0.01 and ***p < 0.001; ns = not significant.

Preclinical therapeutic potency of cancer nanomedicines has been solidly validated, which is always referred to as delaying or inhibiting tumor growth. In almost all cases, tumor is still continuously growing with lagged rate, and we thus believe that tumor growth is delayed rather than inhibited. With the protocol illustrated in Fig. 3A, repeated treatments with LNPs/DOX delay the growth of subcutaneous tumor (Fig. 3B), displaying a significant difference in tumor size (**p < 0.01 *vs* PBS). Unexpectedly, LNPs/DOX fails to demonstrate potency in relieving cancer pain in all the tests, despite an improvement tendency indicated (Fig. 3C-M).

Although it is less neurotoxic than taxanes, platinum compounds, and vinca alkaloids always involved in CIPN, DOX is associated with the induction of oxidative stress and neuroinflammation, which might cause CIPN (Ibrahim Fouad and Ahmed, 2021). Besides, a number of other factors, such as dose intensity, cumulative dose, and therapy duration, also influence the incidence of CIPN, with the mechanisms not fully understood yet (Kacem et al., 2024). In view of this, the unexpected failure of LNPs/DOX in relieving cancer pain might be owing to CIPN induced by repeated LNPs/DOX treatments. To clarify it, healthy mice used to collect BL data received 3-time LNPs/DOX treatments like tumor-bearing mice, and von Frey test as the most sensitive approach was performed. No significant difference is observed compared with BL (data not shown), indicating the absence of CIPN. It is further speculated that following subcutaneous tumor modeling, mice suffer long-term cancer pain, which deteriorates along with tumor growth, thus becoming insensitive to the behavioral tests. This speculation is ruled out by the behavioral profiles receiving the treatment of LNPs/ROP, a pain nanomedicine designed for on-demand and long-term analgesia in our recent work (Sun et al., 2025). As shown in Fig. 3H-M, at 24 h post-treatment of LNPs/ROP, considerable relief of stimulus-evoked pain is observed in all test, which proves that tumor-bearing mice are still sensitive to the tests at this stage. Nevertheless, the behavior of the mice in open field test is not improved following the treatment of LNPs/ROP (Fig. 3C-G), with the mean speed, total distance traveled, time spent in the central area, and distance in the central area remaining decreased like LNPs/DOX group. Taking the efficacy of LNPs/ROP in relieving stimulus-evoked pain into account, this result indicates that behavioral changes in open field test is the outcome of persistent spontaneous pain rather than its real-time manifestation (Zhang et al., 2023). Thus, LNPs/ROP should actually relieve spontaneous pain within its action duration, which is however not enough to alleviate anxiety resulting from persistent spontaneous pain.

The mechanism of cancer pain is quite complex. As cancer pain primarily originates from nervous system, especially peripheral nervous system, rather than tumor site, its treatment is close to the management of chronic non-cancer pain. Although new targeted pharmaceuticals are reported, there are few successful cases in decades, probably due to the dilemma in identifying specific targets in an individual (Chen et al., 2025; Rong et al., 2025; Yuan et al., 2024). Opioids still remain the cornerstone for pain management. Of course, opioids should also be the best choice in this work and however, their acquisition is legally restricted. Local anaesthetics with ROP, bupivacaine, and lidocaine as representatives are a group of medications that block signal transduction in nerve fibers for pain relief through acting on NaV channel, which may be suggested to manage cancer pain when other treatments have failed. ROP, the same family with bupivacaine, shows desired potency and reduced toxicity, and is considered a good alternative to opioids. On the other hand, the duration of action of free ROP is unsatisfactory, which is less than 6 h. Our recent work has revealed that encapsulating it into LNPs significantly extends the duration to at least 24 h, particularly, showing advantages over liposomes (Sun et al., 2025). Such an extended time window will facilitate various behavioral tests. Taking all these factors into account, LNPs/ROP is designed, serving as a positive control of LNPs/DOX.

Fig. 2B-O demonstrate that tumor size is not a major cause of cancer pain. One of the major mechanisms underlying cancer pain is that tumor and immune cells within tumor microenvironment can generate and release substantial algogenic mediators. These mediators sensitize the peripheral sensory neurons and spinal cord neurons, contributing to spontaneous activity and enhanced responsiveness to external stimuli (Mardelle et al., 2024). In such a setting, cancer pain no longer reflects ongoing injury but rather a malfunctioning nervous system. LNPs/DOX treatment merely delays tumor growth, while not significantly altering tumor microenvironment, and therefore fails to relieve cancer pain.

### 3.4 Inhibiting subcutaneous tumor growth can relieve cancer pain

**Fig. 4:**
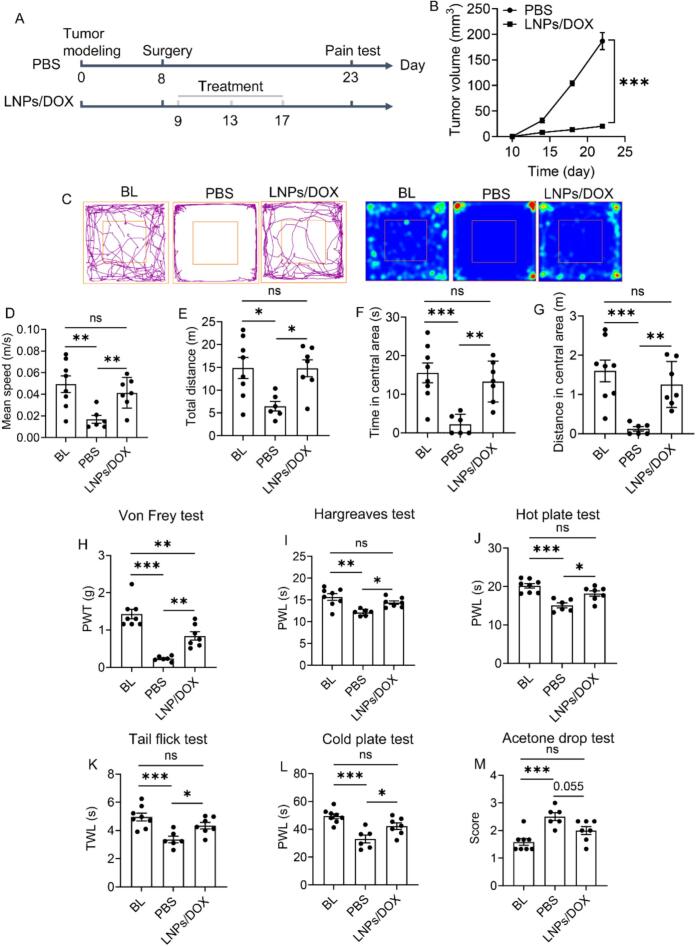
Behavioral test of postoperative tumor-bearing mice receiving PBS and LNPs/DOX treatments. (A) Timepoint for drug treatment and behavioral test. (B) Tumor growth monitoring. (C) Movement trajectory in open field test and corresponding pseudo-color image. (D) Mean speed, (E) total distance, (F) time spent in central area, and (G) distance in central area analyses in open field test. Stimulus-evoked pain evaluation by (H) von Frey test, (I) Hargreaves test, (J) hot plate test, (K) tail flick test, (L) cold plate test, and (M) acetone drop test. Data are presented as mean ± SEM (while *n* = 8 for BL, *n* = 6 for PBS and *n* = 7 for LNPs/DOX, due to the death of mice). BL data come from healthy mice. *p < 0.05, **p < 0.01 and ***p < 0.001; ns = not significant.

It is indeed difficult to inhibit subcutaneous tumor growth if only single nanomedicine-based chemotherapy is applied, no matter whether it is conventional or novel, as fully validated in our previous work. Recently, inhibited tumor growth has been found in a postoperative orthotopic breast cancer model, with tumor growth almost stagnated following LNPs-based chemotherapy (Huang et al., 2024). Thus, we in the following struggle to identify whether tumor growth inhibition can benefit cancer pain relief using postoperative subcutaneous tumor model, with the protocol illustrated in Fig. 4A. Fig. 4B reveals that after surgical removal, tumor is gradually growing and on the 26th day, tumor size reaches 186.8 mm^3^. Similar to non-postoperative subcutaneous tumor (Fig. 3C-M), postoperative subcutaneous tumor also contributes to the generation of severe cancer pain (Fig. 4C-M). As expected, postoperative subcutaneous tumor growth almost stagnates following LNPs/DOX treatment, with tumor size only 20.3 mm^3^ on the 26th day. We thus posit that LNPs/DOX herein inhibits tumor growth. The behavioral profiles further reveal that all pain-related indexes, particularly, exploratory behavior and locomotion activity in open field test, are significantly improved in this case, solidly corroborating that inhibiting subcutaneous tumor growth can relieve cancer pain (Fig. 4C-M). As discussed above that tumor size is not the major cause of cancer pain (Fig. 2B-O), it is strongly suggested that effective tumor growth inhibition relieves cancer pain through altering tumor microenvironment and restoring peripheral and spinal changes. Moreover, it is worth emphasizing that there is a significant difference found between BL and LNPs/DOX group in von Frey test (Fig. 4D), while there is no difference in all the other tests. This result indicates that despite a significant tumor growth inhibitory effect, mice are still undergoing cancer pain, which also validates the high sensitivity of von Frey test.

Clinical causes of cancer pain depend on the histologic type of cancer, its primary location, and its metastatic location (Schmidt et al., 2010). Our findings suggest that the pain causes of subcutaneous tumor in mice might be rather associated with tumor development process. Together with results in other work that cancer pain is prevalent in 100 % of tumor-bearing mice, irrespective of cancer type and location, behavioral test should be a powerful tool to check tumor modeling and evaluate antitumor potency of cancer nanomedicines as well as other drugs. Moreover, as the simplest and the most widely used tumor models, subcutaneous tumor model can serve as an alternative to bone cancer model, the most studied cancer pain model, to study cancer pain.

## 4 Conclusion

We prepare LNP/DOX, a cancer nanomedicine, and LNPs/ROP, a pain nanomedicine, to determine whether therapeutic potency of a cancer nanomedicine can be predicted *in vivo* by pain-related behavioral test in subcutaneous tumor model. Behavioral profiles reveal that subcutaneous tumor modeling in mice can induce both spontaneous and evoked pain, which may deteriorate over tumor growth. LNPs/DOX delays subcutaneous tumor growth, showing a significant difference compared with non-treatment group, and unfortunately fails relieve cancer pain. Using a postoperative subcutaneous tumor modeling, LNPs/DOX inhibits subcutaneous tumor growth and significantly relieve cancer pain in all the behavioral tests. It is thus concluded that pain-related behavioral test can serve as a powerful tool to predict excellent therapeutic potency of a cancer nanomedicine in treating subcutaneous tumor if there is a significant inhibitory effect, which can also be utilized to check subcutaneous tumor modeling. Moreover, von Frey test is suggested the most sensitive to evaluate cancer pain.

## CRediT authorship contribution statement

**Ye Tao:** Writing – original draft, Validation, Methodology, Investigation, Formal analysis. **Xiaohui Cai:** Writing – review & editing, Writing – original draft, Validation, Methodology, Funding acquisition, Formal analysis. **Zhongping Chen:** Writing – review & editing, Writing – original draft, Validation, Supervision, Methodology, Investigation, Funding acquisition, Formal analysis.

## Declaration of competing interest

The authors declare no competing financial interests.

## Data Availability

Data will be made available on request.
